# Determinants Influencing the Electrocardiographic Diagnosis of Left Ventricular Hypertrophy Among Hypertensive Patients

**DOI:** 10.7759/cureus.79217

**Published:** 2025-02-18

**Authors:** Sahasyaa Adalarasan, Yogesh S, Keerthana Prabhakaran, Vishal Rajkumar, S Shivamalarvizhi, Hariharan C

**Affiliations:** 1 Internal Medicine, Madras Medical College, Chennai, IND; 2 Internal Medicine, Madras Medical College and Rajiv Gandhi Government General Hospital, Chennai, IND

**Keywords:** cornell voltage index, ecg (electrocardiogram), hypertension, left ventricular hypertrophy (lvh), social determinant, sokolow-lyon index

## Abstract

Aims and objectives

The present study aimed to evaluate the utility of electrocardiography (ECG) in diagnosing left ventricular hypertrophy (LVH) among hypertensive patients and to compare the diagnostic performance of Cornell voltage product (CVP) and Sokolow-Lyon criteria (SLC) across various demographic and clinical subgroups like age, sex, body mass index (BMI), diabetes, smoking, and alcohol use. The present study was conducted in an Indian population due to the lack of existing studies regarding this topic.

Methodology

The present study is a cross-sectional study conducted at Rajiv Gandhi Government General Hospital, Chennai, involving 544 hypertensive patients divided into "only ECG-positive," "only ECHO-positive," and "both ECHO and ECG-positive" groups. Baseline demographic and clinical data were collected. ECG findings using CVP and SLC were compared with ECHO findings, and statistical analyses were utilized to assess diagnostic sensitivity and subgroup variations.

Results

Older age and male gender were associated with higher diagnostic sensitivity for LVH, while diabetes and obesity were found to reduce sensitivity. Notably, chronic alcohol use emerged as a positive determinant of LVH detection. CVP was more effective in detecting LVH among females, overweight individuals, and alcohol users, whereas SLC was more sensitive among males, smokers, and diabetics.

Conclusions

ECG demonstrates significant potential as a screening tool for LVH in resource-limited settings, particularly when tailored to specific demographic and clinical subgroups. The differential performance of CVP and SLC underscores the importance of personalized diagnostic approaches. Further large-scale studies are needed to validate these findings and optimize the use of ECG for LVH diagnosis in diverse populations.

## Introduction

Left ventricular hypertrophy (LVH) is a cardiac finding characterized by an increase in the mass of the left ventricular myocardium, typically resulting from chronic pressure or volume overload, usually observed in individuals with hypertension. It is a significant risk factor for cardiovascular morbidity and mortality [1]. Identifying LVH in hypertensive patients is crucial for timely intervention and risk stratification in affected individuals.

Echocardiography (ECHO) is considered the gold standard for diagnosing LVH due to its ability to provide detailed structural and functional cardiac assessment; its availability and accessibility remain limited in many parts of India, especially in rural and resource-constrained settings. In contrast, electrocardiography (ECG) is a widely available and cost-effective diagnostic tool, making it a practical option for screening LVH, particularly in a country like India with its diverse healthcare infrastructure.

Numerous studies conducted in European and American populations demonstrate that ECG is known for its high specificity in rejecting LVH [2-4]. Specificity was found to be 97%, 98.8%, and 96.6%, respectively [2-4]. Given the abundance of such studies, the present study focuses on sensitivity rather than specificity. Moreover, there is a noticeable lack of similar research in South Asian or Indian populations. This highlights a crucial question regarding the factors influencing ECG’s effectiveness in diagnosing LVH among Indians, considering potential variations in anthropometric, demographic, and clinical characteristics. Factors such as obesity and gender may contribute to differences in ECG findings.

Among the ECG-based criteria, the Cornell voltage product (CVP) and Sokolow-Lyon criteria (SLC) are widely regarded as the most reliable for diagnosing LVH [5]. Other criteria such as Romhilt-Estes and Framingham's criteria were not chosen because of their unpopularity and point-wise grading system. These criteria leverage ECG’s high specificity to serve as an effective screening tool, identifying individuals who would benefit from further confirmatory testing via ECHO.

This study aims to evaluate the utility of ECG in diagnosing LVH in hypertensive patients in the Indian population and to determine which specific criteria, CVP or SLC, are most suitable for various subgroups. Factors such as age, sex, body mass index (BMI), diabetes mellitus, smoking habits, and alcohol consumption will be analyzed to identify potential variations in the effectiveness of these criteria. This research seeks to optimize the use of ECG as a screening tool and bridge the gap in the current understanding of its applicability to the Indian context.

## Materials and methods

The proposal for the present study was submitted to the Institutional Ethics Committee at Madras Medical College, Chennai, India, and received approval before the commencement of the research (approval number: 58102024, dated October 22, 2024). The data for the present study was sampled from October 24, 2024, to December 24, 2024. Ethical compliance was ensured throughout the study, with written informed consent obtained from all participating patients in their local language. The consent form explicitly outlined the purpose of the study, the procedures involved, and the rights of the participants, including the right to withdraw from the study at any point without any consequences.

The study was conducted at Rajiv Gandhi Government General Hospital, Chennai, India, a tertiary care hospital catering to a large patient base, which facilitated the recruitment of a diverse sample population. The study included 544 hypertensive patients who met the inclusion criteria. Inclusion criteria comprised adult patients diagnosed with hypertension. Patients with pre-existing cardiovascular conditions, congenital heart disease, or incomplete clinical records were excluded in case they were a confounding factor in the pathogenesis of LVH.

The selected patients were stratified into three groups based on their diagnostic findings: "only ECG-positive," "only ECHO-positive," and "both ECHO and ECG-positive" groups. The fourth, the "both ECHO- and ECG-negative" group, was not included in the study as this study focuses more on sensitivity rather than specificity.

For all participants, comprehensive baseline demographic and clinical data were meticulously collected. No particular questionnaires were conducted to verify the self-administered questionnaire but the patient's attender was questioned to ensure maximum validity. This included the patient's name (for author reference only), age, and gender. In addition, key risk factors such as smoking and alcohol consumption were documented based on self-reported histories. The presence of diabetes mellitus was determined using the World Health Organization (WHO) criteria, which define diabetes as a fasting plasma glucose level of ≥126 mg/dL, a 2-hour plasma glucose level of ≥200 mg/dL during an oral glucose tolerance test, or a random plasma glucose level of ≥200 mg/dL in a patient with classic symptoms of hyperglycemia [6]. Glycated hemoglobin (HbA1c) was not included as a diagnostic criterion in the present study. Body mass index (BMI) was calculated using the standard formula, and the patients were subsequently categorized as overweight (BMI ≥ 25 kg/m²) or not overweight (BMI < 25 kg/m²) based on the Asia-Pacific criteria for obesity [7]. These baseline characteristics were essential in evaluating the potential confounding factors influencing LVH detection.

A standard 12-lead ECG was performed for each participant using a calibrated ECG machine with settings of 10 mm/mV and 25 mm/sec. Key parameters related to LVH, such as the CVP and SLC, were specifically recorded. These criteria were chosen as they are widely validated and commonly used in clinical practice for the diagnosis of LVH. The CVP and SLC were calculated using the standard formulae. For patients without prior echocardiographic evaluations, echocardiography was performed using a standard transducer. The procedure was conducted by a trained cardiologist to ensure the accuracy and reliability of the findings. LVH was defined based on either left ventricular systolic dysfunction (LVSD) or left ventricular diastolic dysfunction (LVDD) of any grade, primarily using left ventricular mass index and relative wall thickness. ECHO findings were classified as positive or negative for LVH. Patients with LVH-positive findings on ECHO served as a benchmark group for validating the sensitivity and specificity of the ECG criteria.

All statistical analyses were performed using IBM SPSS Statistics, version 24 (IBM Corp., Armonk, NY). All missing/incomplete data was removed from the analysis and not included in the present study. Descriptive statistics were utilized to summarize the demographic and clinical characteristics of the study population. Continuous variables such as age, BMI, and blood pressure readings were expressed as means ± standard deviations, while categorical variables such as gender, presence of diabetes mellitus, smoking habits, and alcohol consumption were presented as frequencies and percentages.

Descriptive analyses were done to find associations between the criteria and the determinants. These comprehensive statistical approaches aimed to provide a robust evaluation of the utility of ECG in diagnosing LVH within this population. The results obtained were then compared and contrasted with the literature.

## Results

A total of 544 hypertensive patients were included in the study, with a mean age of 49. The patients were divided into three groups: those with both ECG and ECHO findings (n=134), those with only ECG findings (n=148), and those with only ECHO findings (n=262).

Of the total population, 260 (47.79%) were male individuals, and 210 (38.60%) had diabetes mellitus (DM). Chronic smoking habits were reported in 73 (13.42%), while 133 (24.44%) were chronic alcohol users. Approximately 140 (25.74%) of the participants were classified as overweight.

The mean age of patients in the combined group (ECHO and ECG) was 53 years, compared to 47 years in the ECG-only group and 46 years in the ECHO-only group. The proportion of male individuals was highest in the ECHO-only group (125 {47.71%}), while the combined group had a slightly higher proportion of females. Chronic alcohol use was most prevalent in the combined group (37 {27.61%}) and least common in the ECHO-only group (61 {23.28%}). Overweight individuals were predominantly in the ECHO-only group (77 {29.39%})(Table 1).

**Table 1:** Demographics of the present study. ECHO: echocardiography, ECG: electrocardiography, DM: diabetes mellitus, CVP: Cornell voltage product, SLC: Sokolow-Lyon criteria.

Determinant	Total (n=544)	Both ECHO and ECG (n=134)	ECG only (n=148)	ECHO only (n=262)
Age	49	53	47	46
Male	260 (47.79%)	69 (51.6%)	66 (44.9%)	125 (47.71%)
DM	210 (38.6%)	44 (32.56%)	60 (40.53%)	106 (40.46%)
Smoking	73 (13.42%)	18 (13.48%)	17 (11.21%)	38 (14.5%)
Alcohol	133 (24.44%)	37 (27.61%)	35 (23.48%)	61 (23.28%)
Overweight	140 (25.74%)	27 (19.85%)	36 (24.1%)	77 (29.39%)
CVP (M)	2808.04 ± 278	3015.00 ± 264	2851.47 ± 287	2557.64 ± 284
CVP (F)	2064.27 ± 401	2366.67 ± 404	2042.67 ± 398	1783.48 ± 400
SLC	24.99 ± 8.1	26.15 ± 8.0	24.86 ± 8.2	23.97 ± 8.1

The CVP (male-specific) was highest in the combined group (3015.00 ± 264) compared to the ECG-only group (2851.47 ± 287) and the ECHO-only group (2557.64 ± 284). Similarly, the female-specific CVP was highest in the combined group (2366.67 ± 404). The SLC also showed a similar trend, with the highest mean values in the combined group (26.15 ± 8.0) (Table 1).

For the CVP, female individuals comprised 52.13% (n=147) of the study group, with the highest representation in the combined group (84 {62.68%}) compared to 63 (42.57%) in the ECG-only group. Overweight individuals were more significant in the combined group (32 {23.88%}) and least common in the ECG-only group (28 {18.92%}). Alcohol consumption was slightly more significant in the combined group (36 {26.86%}) (Table 2).

**Table 2:** Determinants influencing CVP. ECHO: echocardiography, ECG: electrocardiography, CVP: Cornell voltage product.

Determinant	Mean (n=282)	Both ECHO and ECG (n=134)	ECG only (n=148)
Female	147 (52.13%)	84 (62.68%)	63 (42.57%)
Overweight	60 (21.28%)	32 (23.88%)	28 (18.92%)
Alcohol	72 (25.53%)	36 (26.86%)	36 (24.32%)

For the SLC, male individuals constituted 47.87% (n=135) of the study group, with the lowest representation in the combined group (16 {9.46%}). Chronic smokers and diabetic patients had the highest prevalence in the combined group, at 20 (14.90%) and 38 (28.35%), respectively (Table 3).

**Table 3:** Determinants influencing SLC. ECHO: echocardiography, ECG: electrocardiography, DM: diabetes mellitus, SLC: Sokolow-Lyon criteria.

Determinant	Mean (n=282)	Both ECHO and ECG (n=134)	ECG only (n=148)
Male	135 (47.87%)	16 (11.94%)	119 (80.4%)
Smoking	34 (12.01%)	20 (14.9%)	14 (9.46%)
DM	76 (26.95%)	38 (28.35%)	38 (25.67%)

## Discussion

The demographic characteristics of our study population differ significantly from previous studies conducted in European and American populations. The mean age in our study is 49 years, notably younger than in Western studies, where the mean typically exceeds 60 years, averaging around 67 years [2]. Additionally, our study had a higher representation of female individuals, unlike many studies that report a predominance of male individuals, with a male proportion of approximately 52.2% [8]. Some studies have even been conducted exclusively on male individuals [9]. These demographic differences may contribute to variations in the diagnostic sensitivity and specificity of ECG and ECHO for detecting LVH. Furthermore, previous literature has inconsistently applied standard diagnostic criteria, which could have led to greater variability in the data [10].

Age and gender emerged as key determinants of sensitivity in the present study. Older age groups and male individuals demonstrated higher sensitivity for LVH detection, aligning with prior research [2]. In the combined ECG and ECHO group, the mean age was the highest (53 years), with a male prevalence of 51.60%. Conversely, diabetes mellitus (DM) appeared to reduce sensitivity, consistent with previous literature [3]. DM was more prevalent in the ECG-only group (40.53%), while overweight individuals were more common in the ECHO-only group (29.59%), both factors potentially contributing to reduced concordance between ECG and ECHO findings. This reinforces the notion that metabolic conditions adversely affect LVH detection by masking its electrocardiographic features.

Smoking, as reported in prior studies [4], showed little to no effect on LVH detection in our study. Smokers constituted 13.42% of the total population, with no significant differences across diagnostic groups, suggesting that smoking may not be a major determinant of LVH sensitivity in hypertensive individuals. However, a novel finding in our study is the positive association between chronic alcohol use and LVH sensitivity. Alcohol consumption was highest in the combined ECG and ECHO group (26.86%), suggesting its potential role as a positive determinant in this population. It is also important to note that alcohol-related dilated cardiomyopathy could be a contributing factor in causing ECG changes.

When comparing the performance of CVP and SLC, distinct patterns emerged. CVP demonstrated superior LVH detection in females, overweight individuals, and chronic alcohol users, as evidenced by higher values in these subgroups within the combined group. Conversely, SLC performed better in males, smokers, and diabetic patients. These findings highlight the nuanced performance of these indices across different subpopulations, emphasizing the need for tailored diagnostic approaches.

The present study also identified deviations from existing literature, likely due to population differences [11,12]. For instance, while prior research suggests that obesity universally lowers sensitivity across all ECG criteria, our findings indicate that overweight individuals demonstrated better sensitivity in CVP. Similarly, the positive association between alcohol use and LVH detection represents a novel contribution to the field, warranting further investigation.

It is also important to acknowledge a study conducted on UK Indian Asians, which concluded that this population has a higher likelihood of LVH due to long-standing hypertension [13]. Another study from India highlights clinical strategies for LVH prevention [14]. Additionally, a Cameroonian study produced similar results and conclusions, reinforcing the broader applicability of our findings [15]. Other research has explored the influence of hypertension on left ventricular geometry and volume, providing further insights into the condition’s pathophysiology [16,17].

Limitations

This study faced several limitations that warrant consideration. While the sample size was adequate for preliminary analysis, it was insufficient to fully capture the variability of LVH determinants across subgroups, limiting the generalizability of our findings. This could be attributed to the fact that divergent findings were obtained for smoking and obesity. Observer error in echocardiographic assessments, despite standardized protocols, may have introduced variability in ECHO findings. Future research in the Indian subcontinent with larger cohorts and minimized observer bias is essential to strengthen the clinical applicability of our findings.

## Conclusions

Our study highlights the variability in the sensitivity of ECG and ECHO for diagnosing LVH, influenced by demographic and lifestyle factors. Older age and male gender were associated with higher sensitivity, while DM and obesity reduced it. This aligns with previous research, likely indicating the indifference to ECG determinants seen in Indian and other populations. Chronic alcohol use emerged as a novel positive determinant, while smoking had minimal impact. The nuanced performance of CVP and SLC across different subpopulations underscores the need for personalized diagnostic approaches.

In conclusion, these findings bridge gaps in the existing literature and provide a foundation for more tailored strategies in LVH diagnosis. The usage of CVP among female individuals, overweight individuals, and chronic individuals, and the usage of SLC in male individuals, people with diabetes, and smokers provide higher sensitivity to detect LVH. Future studies should validate our findings in diverse populations, further refining the diagnostic utility of ECG and ECHO in clinical practice.
